# A review of methamphetamine use and stroke in the young

**DOI:** 10.3389/fneur.2024.1397677

**Published:** 2024-04-24

**Authors:** Kafi Hemphill, Shannon Tierney, David Tirschwell, Arielle P. Davis

**Affiliations:** Harborview Medical Center, University of Washington, Seattle, WA, United States

**Keywords:** methamphetamine, stroke, intraparenchymal hemorrhage, young stroke, subarachnoid hemorrhage

## Abstract

Methamphetamine (meth) is a potent and addictive central nervous system stimulant with increasing use. Stroke is one severe possible complication of meth use. Due to high levels of manufacturing in Mexico, the western United States has experienced greater consequences of meth use. The literature reviewed herein is comprised of case studies and series, and it suggests that hemorrhagic stroke (including hypertensive-like intracerebral hemorrhage and aneurysmal subarachnoid hemorrhage), as opposed to ischemic stroke, is the more common type of neurovascular complication of meth use. Meth-related strokes are a particular concern for younger patients with stroke and may be a partial explanation for increasing stroke rates in this age group. We describe two cases (one intraparenchymal hemorrhage and one ischemic stroke) in young patients (<50 years old) with recent meth use to illustrate clinical characteristics and therapeutic considerations. There are several proposed pathophysiological explanations for meth-associated hemorrhagic stroke including an induced hypertensive surge, vasospasm, blood brain barrier breakdown, chronic hypertension, aneurysm development and rupture, and very rarely associated vasculitis. The increased risk of ischemic stroke related to meth use is less well supported in the literature, but this may, in part, be related to a lack of appropriately designed and powered research studies. Proposed mechanisms for ischemic stroke complications of meth use include those affecting blood vessels such as accelerated atherosclerosis, chronic hypertension, vasospasm, and vasculitis, plus mechanisms that affect the heart including cardiomyopathy, arrhythmias, and infective endocarditis (especially with injection drug use). Standard therapeutic interventions for acute stroke and approaches to secondary stroke prevention seem appropriate for meth-associated strokes, with the addition of abstinence from continued meth use. There is no evidence for any meth-specific stroke treatments. Finally, the prolonged duration of meth withdrawal is described. Larger, prospective studies of meth-related strokes are needed to allow for a better understanding and improved care for this often-devastating consequence of an increasingly prevalent cause of strokes in young patients.

## Introduction

Methamphetamine (meth), a potent and highly addictive central nervous system stimulant, is increasingly abused worldwide with far reaching health consequences. Increased risk of stroke is among the most devasting impacts. Escalating rates of stroke in the young have been attributed to a rise in traditional vascular risk factors (1–4), but substance abuse may also contribute (5–9). Given its cardiovascular toxicity (10–13), meth warrants special consideration.

Methamphetamine’s name derives from the additional methyl group on its chemical structure, as compared to amphetamine. This added methyl group enhances lipid solubility, allowing for more rapid transit across the blood brain barrier, increased potency, and longer lasting central nervous system stimulant effects (14–18). The terminal half-life of meth is approximately 10 h with significant variability among individuals due to its hepatic metabolism via cytochrome p450 2D6 (17). Compared to cocaine, which has a half-life of only 0.5–1.5 h, meth has a relatively long effect (19). Once inside the brain, meth increases release and blocks reuptake and degradation of the monoamine neurotransmitters: dopamine, serotonin, and norepinephrine (12, 16, 17).

## Epidemiology of meth use globally and in North America

Worldwide, 1 in 17 people aged 15–64 used a recreational drug in the past year, representing a 23% increase over the last decade. The United Nations drug report describes meth as “the world’s dominant illegally manufactured synthetic drug,” and globally, 36 million people used amphetamines (including meth) (20). In part because it is a synthetic drug that is not dependent on the vagaries of weather, labor, and land as for plant-based drugs, meth is increasingly available. Prevalence of meth use is highest in North America followed by East and South East Asia, but meth is also the prime drug of concern in Australia and New Zealand and is expanding into non-traditional geographic locations including: South West Asia, the Near and Middle East, South East Africa and West Africa (20).

Despite the global expansion of meth trafficking and use, East and South East Asia and North America account for nearly 90% of the global meth seized between 2017 and 2021 with the 2021 seizures of meth in North America at a record high (20). The landscape of North American meth production has shifted over time and in response to legislative attempts to curb meth use and production. Throughout the 1990s and early 2000s, domestic production of meth in the United States (US) rose as both small home-based laboratories and more refined super laboratories were converting ephedrine and pseudoephedrine (found in over-the-counter cold medications) into meth (21, 22). The Combat Methamphetamine Epidemic Act, enacted in 2006 and preceding state legislation, more strictly regulated the sale of meth precursors, substantially suppressing US domestic meth production. However, rather than curtailing meth production and use, this shifted production further into Mexico (23, 24). Today, most meth produced in Mexico is smuggled across the US border, explaining in large part the higher historical prevalence of meth use in the Western US (23, 24). The National Forensic Laboratory Information System compiles drug testing results from participating US state and local labs and provides a sense of regional drug trends over time. In 2022, the majority (39%) of meth drug submissions came from the Western US, compared to 33% in the South, 29% in the Midwest, and 8% in the Northeast (25), suggesting over time that meth has infiltrated beyond the Western US, particularly into the South and Midwest.

In the United States, 1 in 4 people aged 12 or older used illicit drugs in 2022. After marijuana, central nervous system stimulants are the most widely used recreational drug in the US with 10.2 million users, 2.7 million of whom are using meth (26). United States meth use has increased 93% from 2016 to 2022 (26, 27), and it is important to note these numbers do not include some of the highest risk populations—people who are unhoused or in prison. Meth use in 2022 was highest amongst those aged 26 or older (28). The profile of meth users has diversified over time. Looking at US trends from 2015 to 2019, meth users were 50.9% women, and rising use was seen among homosexual men, American Indian or Alaskan Native people, Hispanic people, and White people, as well as those with lower level of education (high school or less) and lower annual household incomes (29).

In addition to meth use increasing and users diversifying, riskier use patterns are emerging in tandem with a rise in overdose deaths. Frequent use of meth (defined by >100 days in the past 12 months) increased from 42 to 50% between 2015 to 2019 (29), and in 2022, around two-thirds of people using meth met criteria for meth use disorder (26). Meth use disorder is defined by the Diagnostic and Statistical Manual, Fifth edition and requires two or more criteria suggesting escalating patterns of use; use regardless of personal, social, or physical harms; and/or signs of tolerance or withdrawal (30). Further escalating concern is the increase in US overdose deaths involving meth (29, 31, 32). Overdose deaths involving psychostimulants other than cocaine (mainly meth) rose 180%, and overdose deaths involving psychostimulants with opiates jumped 266% from 2015 to 2019 (29). This increase in polysubstance overdose deaths with co-involvement of meth and opiates peaked in 2021, the most recent year of data, with 61.2% of deaths due to meth with heroin or fentanyl (31, 32). The trend in co-use of meth with opiates is not limited to overdose; those with meth use are more likely to also have nicotine dependence, cannabis, cocaine, hallucinogen, opioid, and prescription stimulant use (29).

Escalating global use of meth and overdose deaths involving meth create an imperative to improve understanding of its harmful effects, including its cardiovascular impacts. Given the prevalence of meth use in the young (26, 33) and meth’s cardiovascular toxicity (10–13), meth use is highly relevant to stroke in the young. Previous studies, while not specific to meth, have suggested illicit drug use is a relevant contributor to stroke in young adults (6–9). One such study exploring the etiology of stroke in the young identified substance abuse as the fifth most likely etiology following cardioembolism, small vessel disease, hematologic disorders/other, and nonatherosclerotic vasculopathy (8). Depending on the patient population evaluated, stroke risk among substance abusers may be as high as 6.5 times that of non-users (9). Concerningly, there is suggestion that recreational drug use is increasing overall, and higher rates of drug use are being observed in young patients with stroke (6, 7, 9). A more recent look at the Greater Cincinnati Northern Kentucky Stroke registry discovered increased substance use over time among young adults with stroke, rising from 4.4% in 1993–1994 to 28.9% in 2015 (7). While these prior studies of substance abuse amongst the young did not specifically scrutinize meth use, there is a growing body of literature to support the association of meth with stroke in the young.

## Methamphetamine-related hemorrhagic stroke

### Case vignette

**Figure 1:**
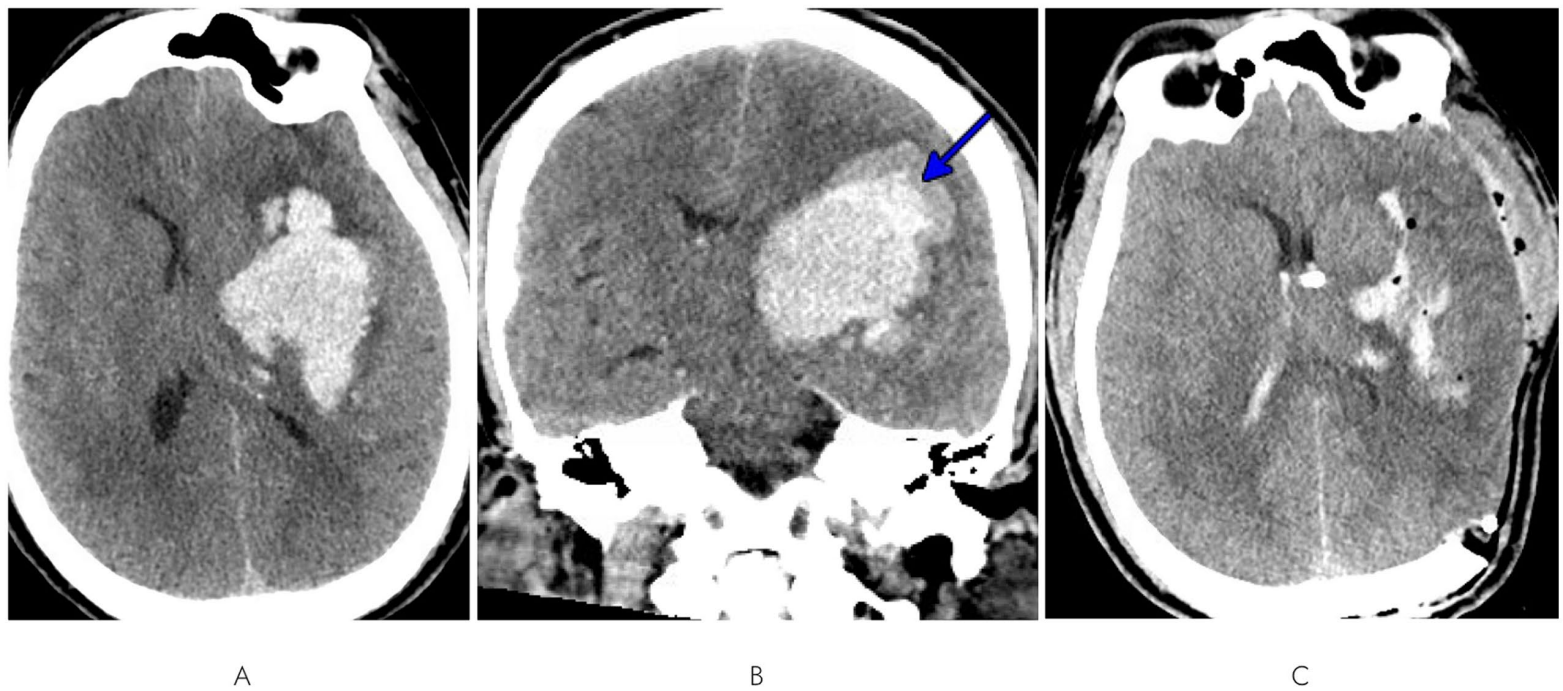
**(A)** Axial and **(B)** coronal views of initial CT head demonstrating a large left basal ganglia intraparenchymal hemorrhage with edema, subfalcine herniation, midline shift, and blend sign (blue arrow). **(C)** Post-craniectomy and EVD CT head displaying hematoma evacuation and corrected midline shift.

A 47-year-old man with a history of untreated hypertension had a fall with seizure-like activity and was found to be aphasic with systolic blood pressures over 250 mm Hg. He was not on anticoagulation but had taken an aspirin earlier that day. He was intubated in the field for airway protection and taken to a local hospital. Computed tomography (CT) head demonstrated a large intraparenchymal hemorrhage in the left basal ganglia measuring 7.4 × 4.5 × 5.1 cm with associated vasogenic edema, subfalcine herniation, and 1 cm of left to right midline shift (Figure 1A). A blend sign was also noted (Figure 1B), suggestive of a high risk of hematoma growth (34). CT angiography (CTA) of the head did not demonstrate any evidence of underlying vascular malformation. A urine drug screen was positive for meth in addition to the benzodiazepines used for intubation. Details on his substance use habits were unclear. He was not on any medications that could have produced a false positive meth result.

A left-sided craniectomy with hematoma evacuation and external ventricular drain (EVD) placement was performed. Post-operative CT head demonstrated successful evacuation of most of the hematoma and correction of midline shift (Figure 1C). A brain MRI was not performed as the etiology was felt to not be in question. He transitioned from intravenous antihypertensives to orals, and normotension was achieved on a two-agent regimen of amlodipine 10 mg daily and lisinopril 20 mg twice daily. He was discharged to a skilled nursing facility 2 weeks after admission with persistent deficits of aphasia and right-sided hemiplegia. Prior to admission, he had been fully independent; at discharge, he was bed-bound with a modified Rankin scale (mRS) score of 4.

### Epidemiology & characteristics of methamphetamine-related hemorrhagic stroke

Of the many adverse effects of meth use, hemorrhagic stroke is one of the best described and most devastating. Since the 1980s, meth use has been linked to subarachnoid and intracerebral hemorrhage (35, 36). A 2017 review found that over 80% of case reports and series on meth-related strokes were hemorrhagic (13). Meth increases the likelihood of intracerebral hemorrhage (ICH) by as much as 2–5 times (11, 37). The incidence of subarachnoid hemorrhage (SAH) among meth users is likely less than ICH. In a 10-year cohort study by Huang et al., the incidence of SAH among meth users was 6.2 per 100,000 person-years compared to 20.8 for ICH (11).

Meth-related hemorrhagic stroke is characterized by distinct patient demographics and hallmark regions of brain involvement. Critically, meth-related hemorrhagic stroke disproportionately impacts a younger population of patients with better pre-morbid functional status and less comorbidities than patients with non-meth-related hemorrhagic stroke (13, 38, 39). Patients with meth-related intracerebral hemorrhage (meth-ICH) tend to be men (13, 39) with lower rates of hypertension and antithrombotic use, but significantly higher rates of smoking (39). Some studies, limited by small sample sizes and selection bias, have suggested racial differences regarding risk of meth-related ICH with potentially higher rates among Pacific Islanders (40) or Hispanic and White people (39).

In the largest single-center cohort of meth-related ICH, meth-ICH and non-meth-ICH were mostly commonly in deep brain locations, but meth-ICH had significantly less lobar involvement (39). This is consistent with conclusions from other case series that meth-ICH tends to occur in deep brain structures, such as the basal ganglia, thalami, or pons—regions classically associated with hypertensive ICH (10, 38, 40). Infratentorial meth-ICH has been less commonly reported than supratentorial meth-ICH (10, 38–42). This discrepancy between supra- and infratentorial meth-ICH mimics that of non-meth hypertensive ICH, in which supratentorial hemorrhages are also more common (43). Increasing numbers of infratentorial meth-ICH are likely to be found in the literature as the total number of cases of meth-ICH rise.

Subarachnoid hemorrhage in patients with meth use is usually aneurysmal (10, 13). Patients with meth-associated aneurysmal SAH (meth-aSAH) tend to be younger than non-users (44, 45). A consensus has not been reached on whether meth-aSAH is associated with a higher rate of vasospasm and worse Hunt and Hess grades (44, 45). In a group of 23 patients with meth-aSAH, aneurysm type (i.e., saccular, fusiform, infectious) and location (anterior vs. posterior circulation, proportion within Circle of Willis) were not significantly different from patients with aSAH but without meth exposure (46). Patients with intravenous administration of meth are also at risk of developing infectious aneurysms, which may indirectly lead to meth-related hemorrhagic stroke, but these patients are rarely mentioned in the literature (47).

### Pathophysiology of methamphetamine-related hemorrhagic stroke

**Figure 2:**
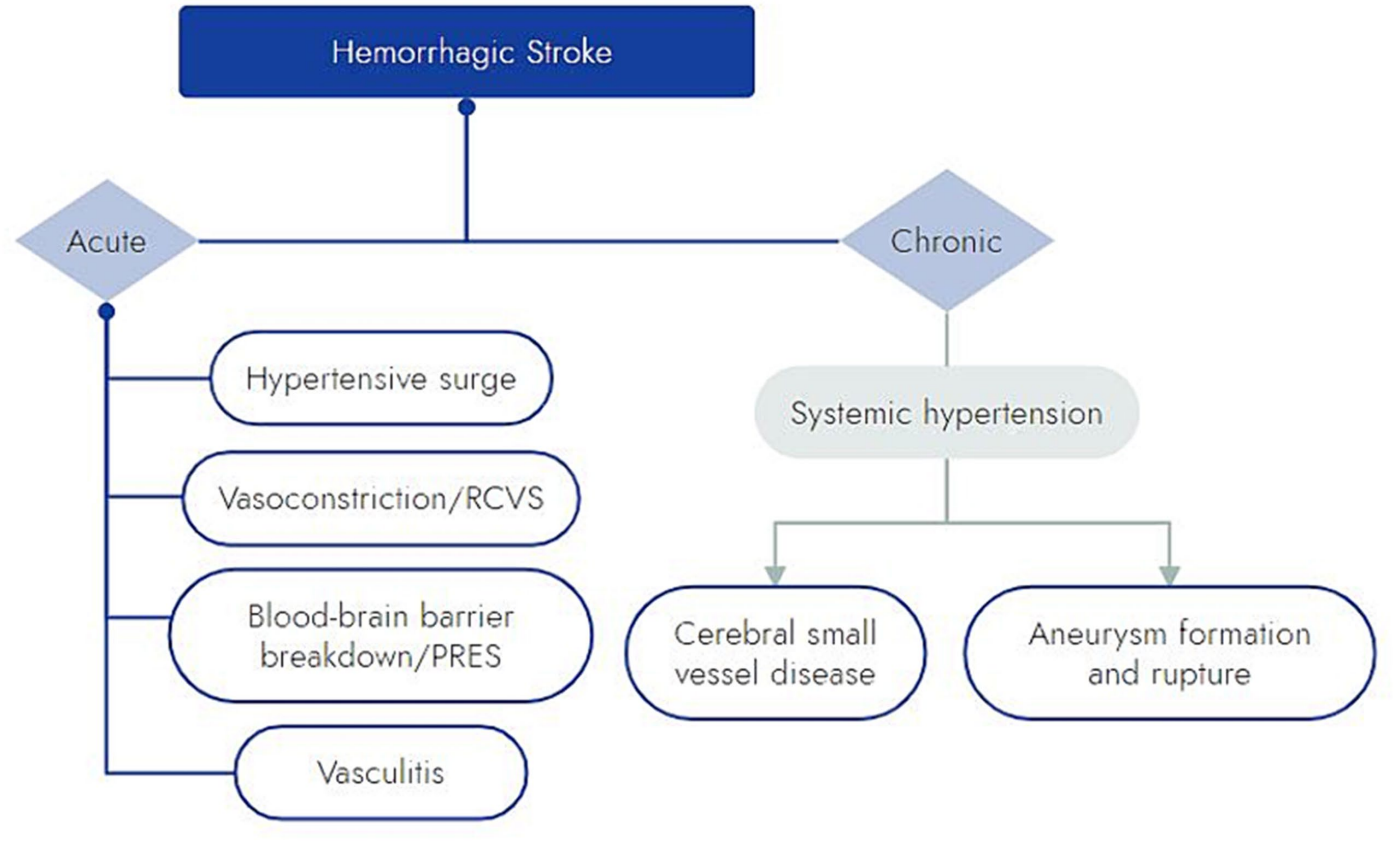
Schematic of proposed mechanisms by which meth may cause hemorrhagic stroke. RCVS, reversible cerebral vasoconstriction syndrome; PRES, posterior reversible encephalopathy syndrome.

The pathophysiology behind meth-associated hemorrhagic stroke remains unclear, but there are several proposed mechanisms (Figure 2). Meth induces a dose-dependent hypertensive surge, which may lead to direct damage and rupture of small penetrating arteries (10, 13, 48, 49). This model is supported by the hypertensive pattern of intracerebral hemorrhage commonly observed in patients with meth-related ICH, even among those without a history of essential hypertension (10, 38–42).

Meth has been shown to induce vasospasm in large and small intracerebral arteries (12, 13, 50). A mouse model has suggested that the endothelin pathway is involved in meth’s vasoconstrictive effect (51). Meth is also known to act upon aminergic receptors like trace amine-associated receptor 1 (TAAR1), which might also play a role in meth-induced cerebral vasoconstriction (12, 52, 53). Despite the well-described physiology of meth inducing vasoconstriction, there are few cases in the literature of hemorrhage due to meth-induced reversible cerebral vasoconstriction syndrome (RCVS), and those that are reported have occurred in the setting of other illicit substances and serotonergic drugs (54).

In addition to acute hypertension and vasospasm, meth induces blood brain barrier (BBB) breakdown (55). In rodents, meth has been shown to induce BBB breakdown within hours of administration (55). BBB breakdown is thought to occur via a combined effect of meth on endothelial cells and inflammatory signaling (55). The role of this inflammatory response in ICH is not clear, but it may exacerbate ICH outcomes or even predispose patients to posterior reversible encephalopathy syndrome (PRES). PRES with hemorrhagic conversion has been reported in a few cases of patients with polysubstance use including meth (56).

Meth-induced vasculitis has also been put forward as a mechanism for meth-induced intracerebral hemorrhage (57, 58). This theory is based on histologic evidence of systemic necrotizing angiitis in 14 patients with intravenous polysubstance use, 12 of whom admitted to the use of meth, and 1 of those 12 with exclusive meth use (59). Only 4 fatal cases were described in detail, 2 with confirmed meth use and 1 with possible meth use. These autopsy cases had histologically confirmed CNS “diseased” arterioles; 1 patient had strokes (both ischemic and hemorrhagic) (59). The diagnosis of vasculitis in these 4 cases was put forth based mostly on findings in other organ systems (59). Thus, despite being heavily cited in the literature, extrapolation from this case series on the potential for meth to induce CNS vasculitis is limited. Several case reports have also linked meth-induced vasculitis with intracerebral hemorrhages and ischemic stroke (42, 60, 61). A study of meth in rhesus monkeys by Rumbaugh and colleagues demonstrated changes in arterial caliber, perivascular infiltration around small arterioles, and microaneurysms, but they did not clearly demonstrate transmural vascular inflammation or necrotizing arteritis (62). In a large series of over 400 deaths in San Francisco in which meth was detected, autopsy did not demonstrate any evidence of necrotizing angiitis (63), and this was further confirmed by an Australian autopsy study evaluating 38 cases of fatal meth-related stroke (64). It is unclear whether meth, other substances, or intravenous contaminates are the true culprits of vasculitis in these case series. Meth-induced vasculitis should be invoked rarely and with skepticism.

With chronic use, meth may increase the risk of intracranial hemorrhage through chronic hypertension, accelerated small vessel disease, and aneurysm formation. Unfortunately, there is a paucity of literature on sustained systemic hypertension related to meth use. In small animal studies, chronic meth use has not been shown to change mean arterial pressure (65). Meth has been well-described as a cause of pulmonary hypertension, and the varied effects of meth on the cardiovascular system make chronic meth use as a driver of systemic hypertension credible (12, 48). Meth use has been associated with increased white matter hyperintensities, independent of other cardiovascular risk factors (66). This is relevant to hemorrhagic stroke pathophysiology as a high volume of white matter hyperintensities has been associated with an increased risk of intracerebral hemorrhage and poorer prognosis (67–69).

Finally, meth-associated aneurysmal SAH (meth-aSAH) has raised interest in the role of chronic meth use in aneurysm development and rupture (10, 13, 70). In a descriptive case series of 62 patients with intracranial aneurysms and meth use by Noblett et al., the median diameter of ruptured aneurysms was only 5.5 mm and the mean 6.3 mm (70). Prior prospective studies reported a risk of rupture of <1% per year rupture in intracranial aneurysms under 7 mm (71–73); thus, Noblett et al. queried whether the meth-aSAH median ruptured aneurysm size of 5.5 mm might reflect a higher rupture risk of small aneurysms for meth users (70). However, a recent meta-analysis of ruptured aneurysms reported a mean size of 6.1 mm, accentuating the limits of size alone in predicting rupture and the importance of other variables such as aneurysm growth, location, and morphology parameters (74). A case of cerebral aneurysm growth over just 3 weeks in a patient with chronic meth use (75) prompts speculation that meth may accelerate aneurysm expansion. The chronic hypertensive and inflammatory effects of meth may foster intracranial remodeling, thereby leading to aneurysm formation (46). Smoking, which is common among meth users, likely also contributes (44, 45). Once aneurysms form, patients with meth-aSAH have similar factors predictive of rupture (76) compared to aSAH without meth, including bottleneck factor (maximum width/neck width) and height-to-width ratio; however, the aspect ratio (a measure of aneurysm height relative to neck width) is significantly larger in the meth-aSAH group (46). Further studies are warranted to clarify the acute and chronic mechanisms by which meth increases hemorrhagic stroke risk.

### Therapeutic considerations & outcomes in meth-related hemorrhagic stroke

As illustrated by the case above, the presence of meth does not demonstrably change the acute management of hemorrhagic stroke. There has been a historical reluctance among clinicians to use beta-blockers in patients with stimulant use due to a theoretical risk of unopposed alpha receptor stimulation exacerbating vasoconstriction, but real-world evidence is lacking (77). Surgical management of meth-related hemorrhagic stroke, including EVD placement, minimally invasive clot evacuation, decompressive craniectomy, and intervention on aneurysms, should be pursued according to the most up-to-date evidence among non-meth users. There is a significant knowledge gap on the surgical management of meth-related aneurysms. Prospective studies are required to clarify the risk of aneurysm rupture in meth users, particularly as it relates to aneurysm size, location, and rate of growth.

The effect of meth use on outcomes after hemorrhagic stroke remains uncertain. In a retrospective analysis by Swor et al., meth-positive patients with ICH (n = 41) were compared to meth-negative patients (38). Meth-positive patients had a higher mean arterial pressure, higher diastolic blood pressure, required more days of IV hypertensive agents, and had longer ICU and hospital stays (38). In a similar but slightly larger study (*n* = 61 meth-ICH), there was no significant difference in mean blood pressures, duration of ICU care, or length of hospital stay between patients with meth-ICH and non-meth-ICH (39). There was also no significant difference in NIHSS, GCS, or ICH scores between groups, though patients with meth-ICH were more likely to undergo surgical intervention, which might be explained by their younger age (39). At discharge, there was no significant difference in functional outcomes or mortality between the meth and non-meth ICH groups, but the change from pre-morbid to discharge mRS was greater in the meth-ICH group (38, 39).

Meth has been hypothesized to worsen outcomes in aSAH. Beadell et al. reported worse outcomes at discharge in patients with aSAH and meth use compared to age-matched controls (median Glasgow Outcome Score 3 vs. 5, *p* < 0.001) (44). Meth use has also been associated with worse clinical outcomes from aSAH at 1 year (OR = 5, 95% CI 1.03–24) and 3 years (OR = 7.2, 95% CI 1.2–30) compared to non-meth users (45). These retrospective studies suggest an association of meth use and poor outcomes from aneurysmal SAH but are hampered by small sample sizes, selection bias, high rates of polysubstance use among meth users, and a lack of reporting on the dose and duration of meth exposure.

## Methamphetamine-related ischemic stroke

### Case vignette

**Figure 3:**
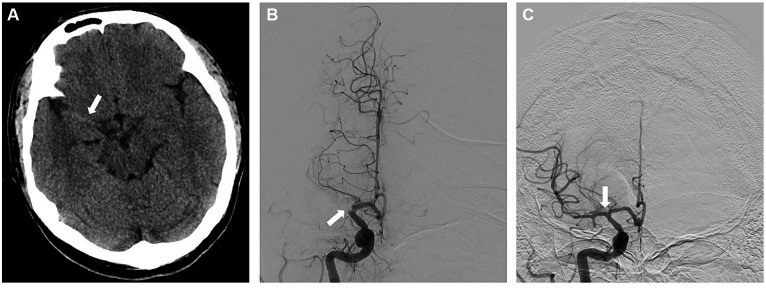
**(A)** Non-contrast CT showing the right-sided hyperdense vessel sign in the MCA. **(B)** AP view of the right ICA injection on the digital subtraction angiogram (DSA), showing the proximal right M1 cutoff. **(C)** AP view of the right ICA injection following successful reperfusion, early arterial phase.

**Figure 4:**
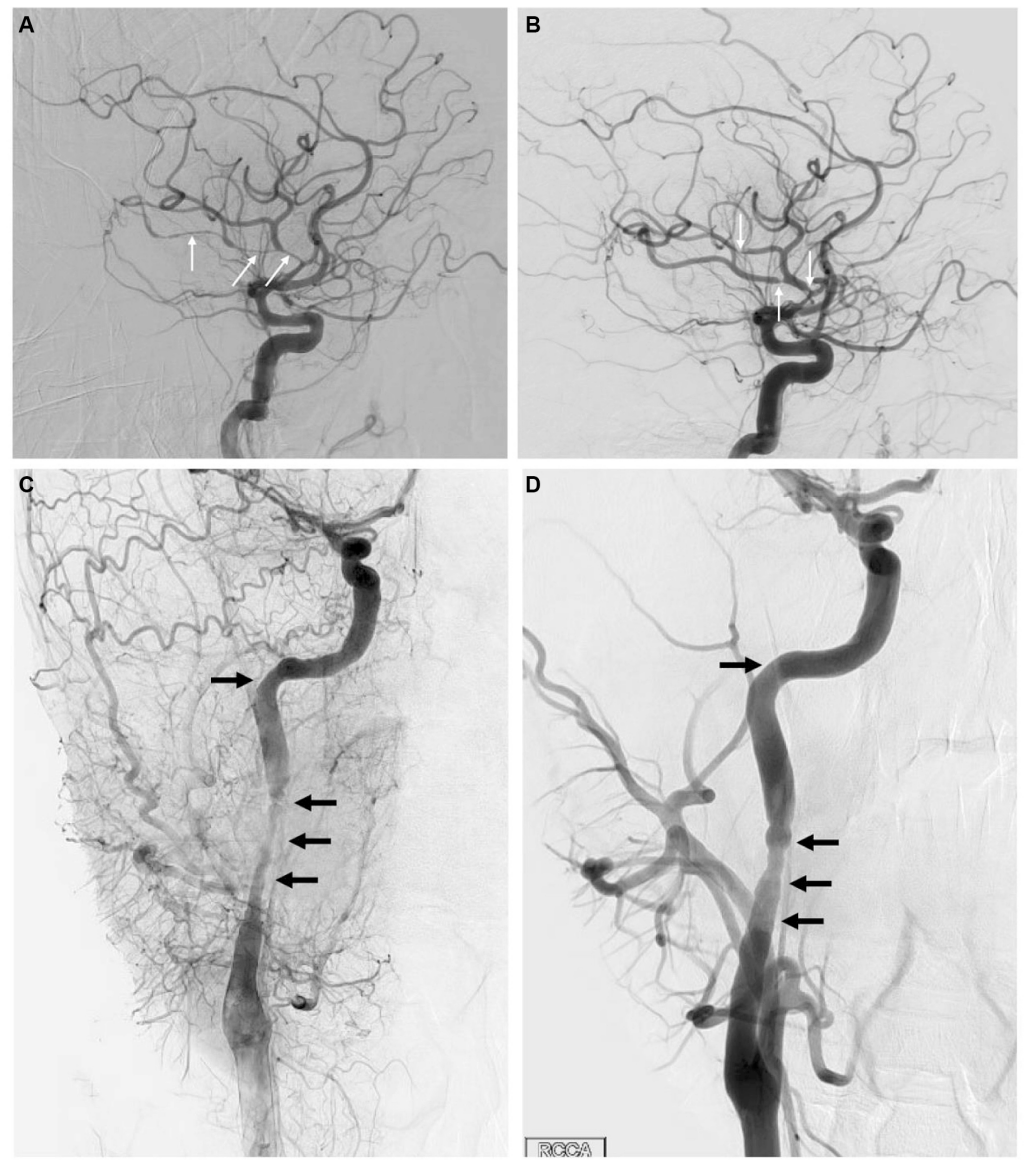
**(A)** Lateral view of the right ICA injection on DSA, showing multiple areas of vasospasm within the MCA branches (arrows). **(B)** Same view following milrinone treatment, showing significant improvement in vasospasm with much shorter affected segments (arrows). **(C)** Lateral view of the cervical right ICA through a right CCA injection on DSA in the late arterial phase, showing significant vasospasm (arrows) within the proximal right ICA. **(D)** Same view following milrinone treatment, showing significant improvement in vasospasm (arrows).

A 33-year-old man presented within an hour of symptom onset with sudden left hemiparesis, right gaze deviation, and confusion. Initial NIH Stroke Scale (NIHSS) was 15. CT head showed a slightly hyperdense right M1 (Figure 3A), confirmed to be a right middle cerebral artery (MCA) occlusion on vessel imaging. He was given tenecteplase at a local hospital, then transferred to a comprehensive stroke center for thrombectomy. Digital subtraction angiogram (DSA) revealed a persistent right M1 occlusion as well as a right A2 occlusion, but access was complicated by significant vasospasm with instrumentation of the internal carotid artery (ICA) (Figure 4C). After 1 pass with combined stent retriever and aspiration approach, there was successful TICI 2b reperfusion (Figures 3B,C). However, beyond the area of occlusion, multiple foci of vasospasm were noted in the MCA territory (Figure 4A). The patient was treated with 3 mL of intra-arterial (IA) milrinone for the MCA vasospasm. The balloon catheter was then retracted, and 5 mL of IA milrinone were given for the ICA vasospasm. After milrinone treatment, there was angiographic improvement of the vasospasm (Figures 4B,D).

Urine toxicology screen was positive for meth, but no additional history of use was documented. Other stroke work up returned unremarkable with normal transthoracic echocardiogram (ejection fraction 65%, normal atrial and ventricular size, no interatrial shunt), no atrial fibrillation on 2 days of inpatient telemetry, LDL 48, hemoglobin A1c 5.5, BNP 33, and minimal atherosclerotic disease on CTA. Long-term cardiac monitoring was not performed. MRI Brain on hospital day 3 showed a moderate sized stroke involving the basal ganglia, insular cortex, and temporal lobe with minimal petechial hemorrhage. Fazekas score was 1 with periventricular caps but no deep white matter hyperintensities outside of the acute stroke. The etiology of stroke was felt secondary to meth-induced vasospasm. His course was complicated by headaches, which were treated successfully with gabapentin. A repeat CTA on hospital day 2 did not show evidence of continued vasospasm, so no vasodilatory medications were used. On hospital day 9, he was discharged to inpatient rehab, where he stayed for 1 month. At discharge from rehab, the patient could walk independently with a single point cane, though he continued to have significant left hand and finger weakness. He was treated with aspirin 325 mg daily and tizanidine for post-stroke spasticity.

### Epidemiology of methamphetamine-related ischemic stroke

While not as common as hemorrhagic stroke, ischemic stroke has been reported as a sequela of both recent and chronic meth use in multiple case series (10, 13, 64, 78). The incidence of meth-associated ischemic stroke is unknown and represents a current knowledge gap. Two population-based studies report a lack of statistical association between meth use and ischemic stroke (11, 37). It must be noted that both studies identified their cohorts using ICD codes for hospitalized patients, which introduces bias in patient selection. Huang et al. (11), who conducted a 10-year follow up study in Taiwan with a meth cohort, posited that an even longer duration of monitoring may be needed to see the association between meth use and ischemic stroke.

In terms of stroke subtypes, Zhu et al. (66) compared meth and non-meth users with ischemic stroke admissions at a single center in California and found no significant difference in the percent of strokes from small vessel disease (31% vs. 28%), large vessel disease (25% vs. 24%), or cardioembolism (34% vs. 46%). They did, however, find increased burden of microvascular ischemic disease on MRI in meth users compared to a propensity matched control group (66).

Route of administration is rarely reported in the literature, so it is unclear if this has any impact on the risk of neurovascular disease. In a small case review of 17 ischemic strokes, patients with inhalational use represented 4 times the number of cases compared to oral use or injection use (13). Further investigation is warranted as the small case numbers in these studies increase the risk of bias.

### Pathophysiology of methamphetamine-related ischemic stroke

**Figure 5:**
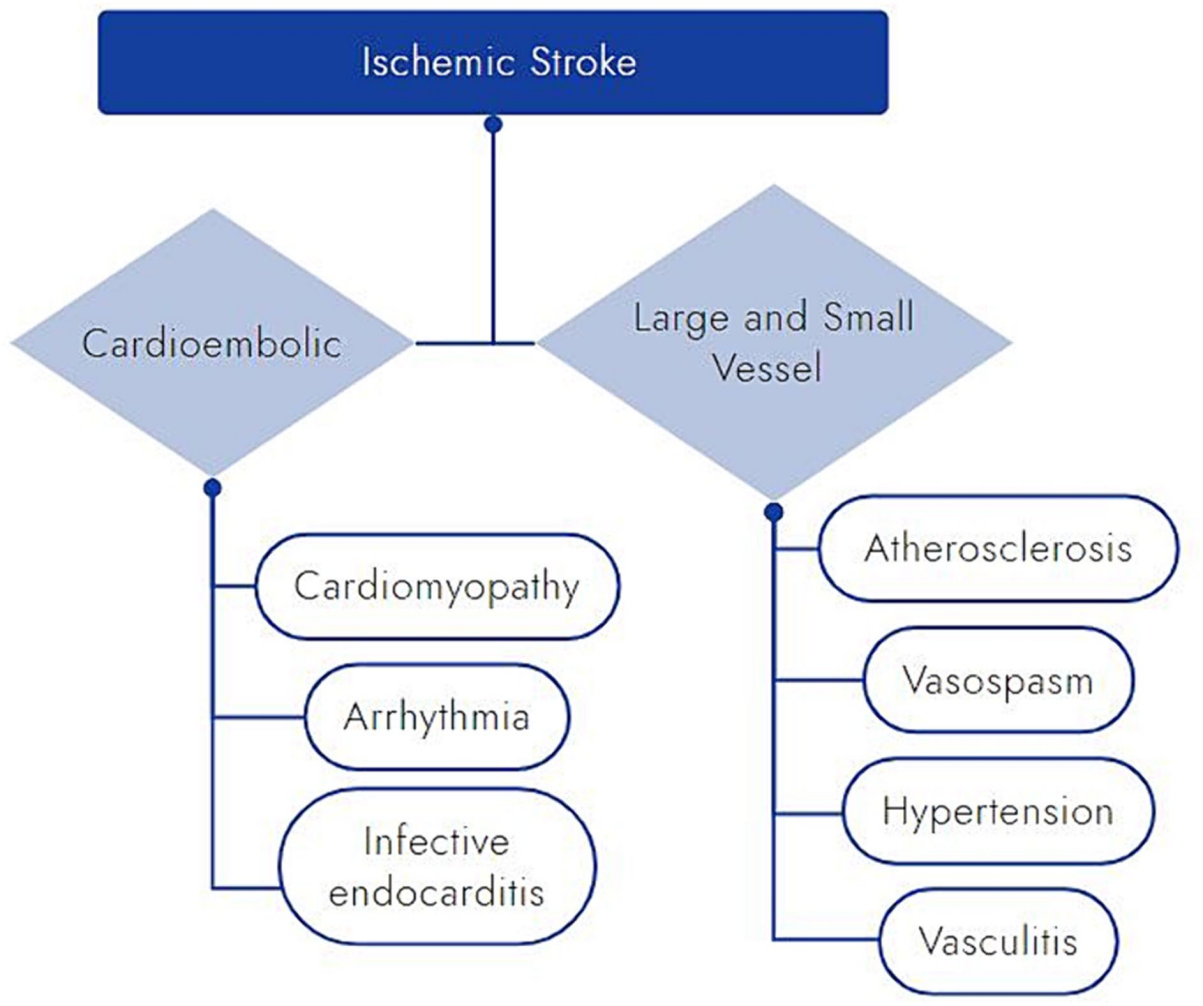
Schematic of proposed mechanisms by which meth may cause ischemic stroke.

Despite the paucity of epidemiologic evidence for meth-associated ischemic stroke, there are multiple pathophysiologic pathways by which meth use may contribute (Figure 5). As already discussed apropos to hemorrhagic stroke, meth has direct effects on blood vessels, leading to hypertension, acute vasospasm, and rarely vasculitis (12, 13, 50, 57–59). Unique to ischemic stroke pathophysiology, chronic meth use also accelerates the development of atherosclerotic disease in both large and small vessels (10, 13, 64, 79). In a mouse model, Gao et al. showed a dose-dependent increase in aortic atherosclerotic disease after 24 weeks of meth use without any change in the circulating lipids, prompting suspicion for an alternative explanation (79, 80). Meth appears to act through multiple proinflammatory mechanisms, including increased production of reactive oxygen species (12, 81). These result in endothelial activation and smooth muscle fibrotic remodeling, which can lead to overall increased atherosclerotic plaque burden as well as potentially increased plaque vulnerability in chronic meth users (12, 82).

The vascular effects of meth can also lead to cardioembolic strokes. In animal models, long-term meth exposure results in cardiomyocyte dysfunction and diffuse edema through a combination of inflammatory and ischemic mechanisms, which in turn, lead to fibrosis, necrosis, and enlargement of the heart (12). Meth-associated cardiomyopathy is typically characterized by isolated left or bilateral ventricular enlargement and reduced ejection fraction, and it can lead to ischemic stroke via several mechanisms. In a small series of 30 meth users with cardiomyopathy and reduced ejection fraction, 33% (10/30) of patients were found to have ventricular thrombi (83). Meth cardiomyopathy also disrupts electrical conduction, leading to arrhythmias. QTc prolongation was the most frequent electrocardiogram abnormality at 27% in a cohort study of 158 meth users (84). Atrial fibrillation is a major source of cardioembolic strokes, and a 2022 database analysis of California residents showed meth users had an 86% increased risk of atrial fibrillation diagnosis, as compared to their non-user counterparts (85). Importantly, the cardiotoxic effects of meth have been documented with acute, chronic, and binge-pattern meth use, but the severity of use is an independent predictor of outcomes (12, 86–88).

Infective endocarditis (IE) is another possible etiology for ischemic stroke in people using intravenous meth. Reports of resultant stroke are difficult to find in the literature, but Johnstone et al. used a Canadian cohort to compare IE patterns for people who inject opioids and stimulants. They found that, while 66% of opioid users with first-time IE developed a right heart infection, there was an even distribution of left and right-sided heart disease among stimulant users (75% meth but also included cocaine, buproprion, and methylphenidate), creating increased potential for embolic stroke (47).

### Therapeutic considerations in methamphetamine ischemic stroke

Unsurprisingly, given the low number of cases, clinical outcomes following acute therapies for meth-associated ischemic stroke are not well-reported in the literature (89). There is only one case report documenting the use of thrombolytics. McIntosh et al. described the case of a young woman with recent meth use who presented with a left ICA dissection and an NIHSS of 21. She received IV alteplase (tPA) within 80 min of symptom onset and had significant improvement in symptoms with follow up MRI showing a small left frontal lobe infarct (90). A single center review of 29 cocaine-positive patients showed no complications of tPA use for acute strokes, despite these patients having more severe stroke symptoms at baseline than their cocaine-negative comparator group (91). While avoiding the delay of time-sensitive therapies, it may be appropriate to discuss the lack of outcome data among patients with meth use with the patient or family members prior to thrombolytic administration, if substance use history is known.

Thrombectomy is similarly infrequently discussed in the literature. Chapman et al. described a young man with 6 months of sustained meth use and a resultant severe cardiomyopathy who presented with a cardioembolic right MCA occlusion. He had a technically successful mechanical thrombectomy 5 h after symptom onset and clinical resolution of neurologic deficits (92). No further details of the thrombectomy were given. Borrowing from the cardiovascular literature, Khaheshi et al. asserted that consideration of aspiration thrombectomy, balloon angioplasty, and/or medical therapies as first line therapies is appropriate when vasospasm or atherosclerotic disease are the favored mechanisms of vessel occlusion (93). Loewenhardt et al. described a chronic meth user who presented with an MCA occlusion that was treated with balloon angioplasty without complication (78, 94). Outside of the case presented in this paper, there is no mention of vasospasm as a complication of angiographic interventions in the literature.

In summary, there is a lack of high-quality data on acute stroke therapies in meth users. However, it makes clinical sense to offer these patients standard therapies, including thrombolytics or thrombectomy if they otherwise qualify. The same principle applies in choosing an appropriate antithrombotic regimen for secondary stroke prevention. Providers should be vigilant against any negative bias related to substance use that might influence stroke management decisions.

## Diagnostic challenges and management

Clinical suspicion and appropriate testing are critical for the recognition of meth use in young stroke patients. Urine toxicology can detect meth up to 4 days following last use, but amphetamines are also the most reported false positive result (95). Due to cross-reactivity, use of certain antihistamines and decongestants (brompheniramine, phenylpropanolamine, nasal inhaler, ranitidine), antidepressants (buproprion, trazodone), and antipsychotics (chlorpromazine, promethazine) can result in false positives on initial screening tests (95). Importantly in the care of stroke patients, a metabolite of labetalol can also produce a false positive for amphetamines (96). Typically, confirmatory testing with liquid chromatography-mass spectrometry (LC–MS) can distinguish false positives from true positives (97). Urine tox screening also misses chronic or binge meth users who have not used in the last 4 days, so there is no substitution for taking a thorough substance use history.

Substance use history should include route of administration, frequency/duration of use, and co-administration with other drugs, but considering the significant stigma that comes with substance use, this can be a challenging history to obtain. Coffin et al. recommend a motivational interviewing style to assess the patient’s understanding of the benefits and harms of meth in addition to their goals regarding future use (98). Zhu et al. (39) found that, after excluding those with recent exposure to medications that can lead to false positives, 7.7% (29/379) of patients in a hemorrhagic stroke cohort who denied meth use tested positive for amphetamines on a urine drug screening. Clear communication may be another barrier to accurate history taking, as meth is known by many street names, including crank, ice, crystal, speed, and glass (99). An understanding of the local terminology may improve accuracy.

Meth cessation is a cornerstone of optimal patient care. Existing studies provide moderate-strength evidence that meth cessation is associated with reversal of cardiac dysfunction, left ventricular remodeling, and improvement in NYHA functional class (100). Reduced exposure to the sympathomimetic effects of meth likely results in better blood pressure control. It would be reasonable to conclude that stroke risk decreases with cessation of meth use, though this is not clearly documented within the literature.

There are no proven pharmaceutical options for meth cessation. Mirtazapine has shown some promise in phase 2 trials for the reduction of meth use and of sexual risk behaviors that can increase risk of HIV infection, but additional research on larger populations is needed (101, 102). Behavioral therapies are the standard of care with the strongest evidence in support of contingency management, a therapy that reinforces abstinence through incentivization (98). In AshaRani et al. (103) review of 44 studies, contingency management outperformed cognitive behavioral therapy (CBT) in selected studies, but used alone, both therapies were effective during treatment periods. There is limited data for sustained abstinence after these therapies. In a sample of 350 patients admitted to treatment centers in Los Angeles, Brecht & Herbeck found a relapse rate of 61% within the first year (104). Unsurprisingly, the strongest predictive factor for prolonged time to relapse is the patient’s engagement in self-help and/or additional treatment during the period of abstinence. Addiction medicine and social work should be engaged in the care of these patients and optimally connect them to outpatient resources for long-term abstinence.

An oft overlooked complication of hospitalization or voluntary cessation is the prolonged withdrawal from meth. Withdrawal is characterized by an “early crash” (12–24 h) with exhaustion and fatigue followed by the “withdrawal phase,” featuring depressive symptoms with increased sleeping and eating (2–4 weeks). Following this, the “extinction phase” can last up to 6–12 months with continued cognitive deficits and mood changes (105, 106). When this withdrawal period overlaps with stroke recovery, it can be difficult to distinguish the etiology of new mood and mental status changes.

## Conclusion

The rise of meth use increases the importance of clarifying the role of meth in stroke physiology and outcomes, especially since meth-related stroke has its greatest impact on the young. Meth poses a clear risk of hemorrhagic stroke and is described as a factor in ischemic stroke. Proposed mechanisms of meth-related hemorrhagic stroke include hypertension, vasoconstriction (rarely) inflammation/PRES and vasculitis, and aneurysm formation and rupture. Chronic hypertension, vasospasm, vasculitis, accelerated atherosclerosis, and cardiac toxicity may precipitate meth-related ischemic stroke. By virtue of the younger age of this population, patients with meth-related stroke have a greater increase in disability than non-meth users. Standard stroke therapies, with the addition of support for meth-cessation, should be employed. Recognition of meth use in young patients with stroke can be challenging as it requires prompt suspicion, urine testing, and a thorough history on substance use habits. Unfortunately, meth is becoming increasingly relevant to stroke in the young, hastening the urgency for further investigation and higher quality prospective studies.

## Author contributions

KH: Writing – original draft, Writing – review & editing. ST: Writing – original draft, Writing – review & editing. DT: Writing – original draft, Writing – review & editing. AD: Writing – original draft, Writing – review & editing.
